# Foundation Models for Microbiome Research: From Sequence Semantics to Community Dynamics and Multimodal World Models

**DOI:** 10.1002/ggn2.70046

**Published:** 2026-09-04

**Authors:** Haohong Zhang, Zixin Kang, Kang Ning

**Affiliations:** ^1^ Key Laboratory of Molecular Biophysics of the Ministry of Education Hubei Key Laboratory of Bioinformatics and Molecular‐Imaging Center of AI Biology Department of Bioinformatics and Systems Biology College of Life Science and Technology Huazhong University of Science and Technology Wuhan Hubei China

**Keywords:** artificial intelligence, bioinformatics, microbiome

## Abstract

Microbiome sequencing has advanced faster than microbiome understanding. Although large‐scale 16S, metagenomic, metatranscriptomic, and proteomic datasets have accumulated rapidly, most analyses remain cohort‐specific and association‐driven, limiting mechanistic insight, cross‐study transferability, and robustness to technical confounding. Foundation models offer a new computational framework by learning reusable biological representations from large unlabeled datasets. In this Review, we present microbiome foundation models as a hierarchy spanning biological scales. Sequence‐centric models capture the syntax and semantics of DNA and proteins for taxonomic inference, functional annotation, and generative design. Community‐centric models learn ecological structure from abundance profiles, while addressing compositionality, sparsity, and the unordered nature of microbial communities. Emerging multimodal frameworks integrate sequence‐derived functional potential with community‐level ecological dynamics under host and environmental context. We discuss key design choices, including tokenization, representation granularity, self‐supervised objectives, and evaluation strategies, and highlight challenges in interpretability, domain shift, causal reasoning, and biological validation. Finally, we propose a transition from static representation learning toward intervention‐aware microbiome world models capable of simulation, digital twinning, and generative microbiome engineering.

## Graphical abstract

Microbiome foundation models are framed as a multiscale route from sequence semantics to community ecology, multimodal alignment and future world models. By learning transferable representations across cohorts, habitats and perturbations, this roadmap highlights how microbiome AI can move beyond association‐driven analysis toward interpretable prediction, intervention reasoning, digital twins and microbiome design.

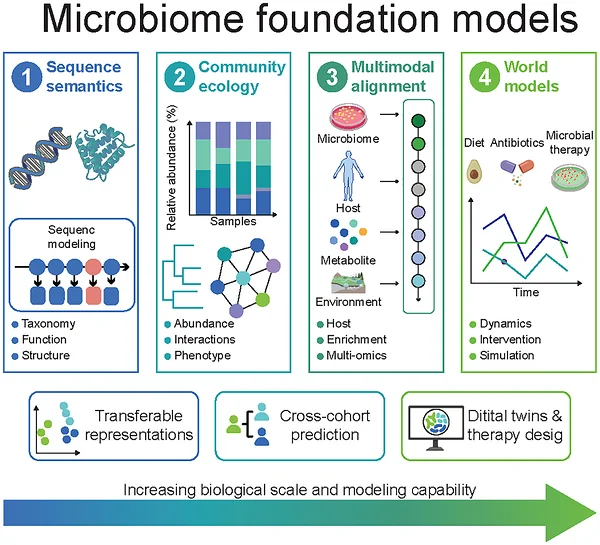

## 1 Introduction

Microbiome research now operates in a paradoxical regime: sequencing has scaled rapidly, but biological understanding has not advanced at the same pace [1, 2]. Over the past decade, 16S rRNA profiling, shotgun metagenomics, metatranscriptomics and related assays have produced a vast observational record of microbial life across hosts, habitats and perturbational states [3, 4, 5, 6]. Nevertheless, the dominant analytical pipeline remains largely cohort‐bound and association‐based [7, 8]. Differential abundance methods [9, 10], ecological summary statistics [11, 12] and classical machine learning [13, 14] have been effective at identifying taxa associated with disease, lifestyle or environmental gradients within individual datasets, yet far less successful at building representations that survive technical shift, ecological variation and annotation incompleteness.

This limitation reflects more than insufficient model capacity. It arises from a mismatch between microbiome biology and the assumptions of conventional analytical frameworks [15]. Microbiome data are sparse, compositional, hierarchical, and deeply context dependent [16, 17, 18]. They span multiple levels of organization, from nucleotide motifs and proteins [19] to genes and operons [20], genomes and strains [21], taxa and communities [22], and host‐linked physiological states [23]. The same taxon can support distinct ecological roles under different environments, while similar community phenotypes can arise from different genomic substrates [24]. In such systems, the central challenge is not merely to identify signal, but to learn abstractions that remain meaningful across scales and domains.

Foundation models offer a qualitatively different response [25]. Rather than fitting a task‐specific predictor to a single labeled dataset, they are pretrained on large‐scale, largely unlabeled corpora to learn reusable representations that can support diverse downstream tasks [26, 27]. In microbiome research, this changes the central question. The issue is no longer simply whether a microbe is associated with a phenotype in one cohort, but what microbial sequences encode, how communities organize, how host and environmental context modulate function, and how these layers combine to shape phenotype [28, 29, 30]. The promise of microbiome foundation models therefore lies not only in improved prediction, but in a shift from descriptive association toward representation, generation, and intervention‐aware inference [31].

**FIGURE 1:**
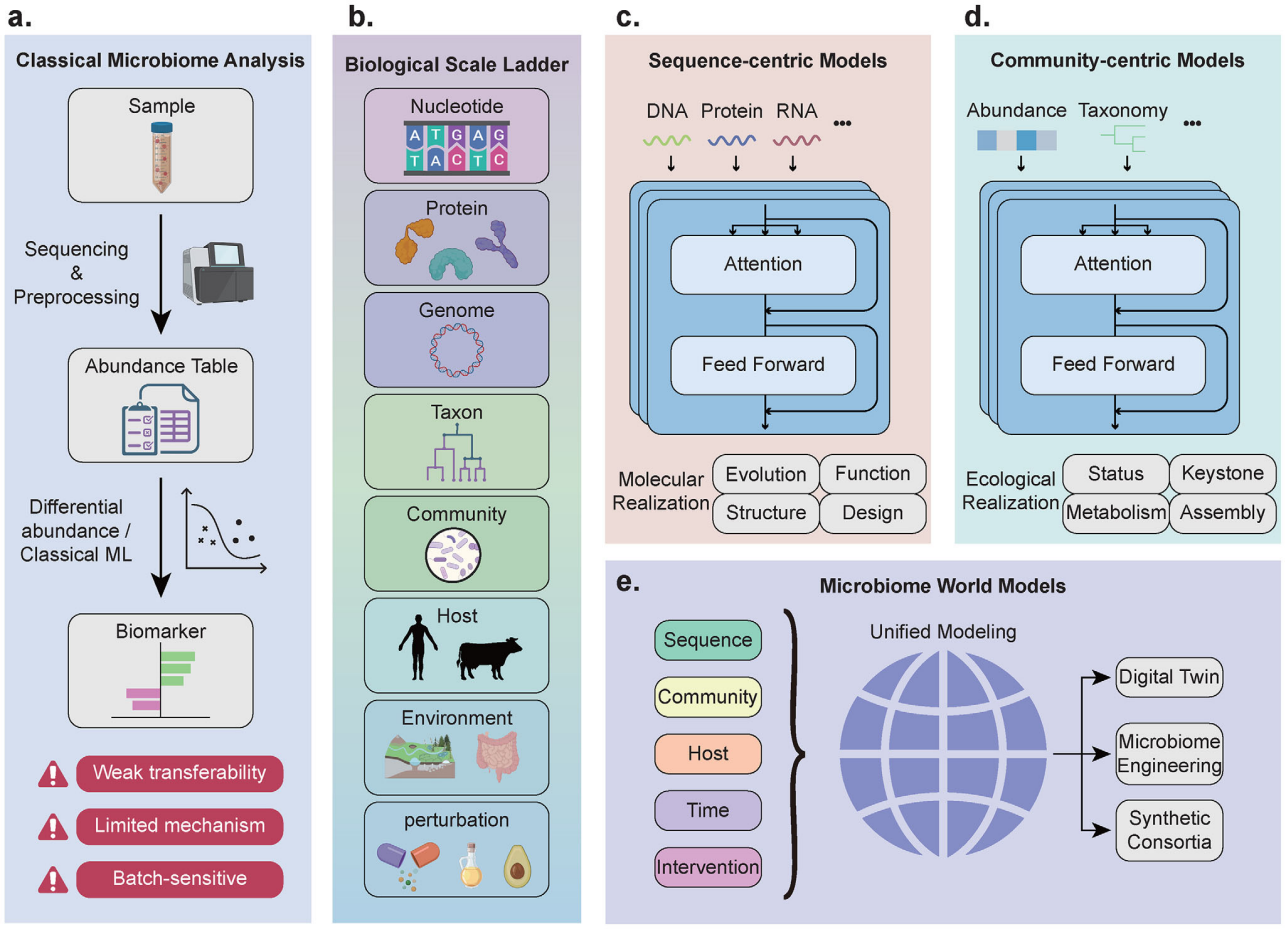
Microbiome foundation models across biological scales. (a) Classical microbiome analysis relies on cohort‐specific abundance tables and statistical learning to identify biomarkers, but is often limited by weak transferability, batch effects, and limited mechanistic interpretability. (b) Microbiome intelligence is organized across biological scales from relatively simple molecular units to increasingly complex system‐level states, progressing from nucleotide and protein sequences to genes and genomes, taxa and communities, host context, and finally environmental and perturbational dynamics. Representation challenges increase with biological scale and system complexity. (c) Sequence‐centric foundation models learn functional potential from DNA and protein sequences through self‐supervised learning, enabling taxonomy inference, functional annotation, structure prediction, and generative design. However, these models primarily represent molecular capability rather than realized ecological activity and therefore often lack sufficient host and environmental context. (d). Community‐centric models learn ecological state representations from abundance profiles despite the absence of natural sequential structure. Emerging multimodal models align sequence‐derived functional potential with ecological realization by integrating community composition, host state, and environmental context. (e) Future microbiome world models may integrate sequence, community, host, and temporal information to enable intervention‐aware prediction, digital twins, and rational microbiome engineering.

In this Review, we argue that microbiome foundation models are most productively understood as a family of approaches distributed across biological scales (Figure 1). Sequence‐centric models operate on DNA or protein strings and aim to capture evolutionary, structural, and functional regularities. Community‐centric models operate on abundance or composition profiles and aim to capture ecological organization, interaction structure, and phenotype‐linked variation. Multimodal models seek to align these levels with host, environmental, spatial, temporal, and perturbational context. Taken together, these paradigms point toward a longer‐term destination: microbiome world models capable of simulation, perturbation reasoning, intervention planning and design.

## 2 Contribution and Scope of This Review

Several recent reviews have summarized the broader use of artificial intelligence and machine learning in microbiome research, including applications in disease prediction, multi‐omics integration and microbiome‐based intervention design [28, 30]. However, these reviews have largely considered foundation models as part of a broader computational toolkit, rather than asking what makes a foundation model specifically microbiome‐relevant. This leaves an important conceptual gap: the field still lacks a scale‐aware framework for distinguishing genuinely transferable microbiome foundation models from large task‐specific predictors trained on microbiome data, and for evaluating whether their learned representations remain biologically meaningful beyond the cohort, habitat, or assay context in which they were developed.

This Review fills this gap by organizing microbiome foundation models according to the biological scale at which representation learning occurs. We distinguish sequence‐centric models that learn molecular syntax and functional potential, community‐centric models that learn ecological state from abundance or composition profiles, and multimodal models that attempt to align these layers with host, environmental, spatial, temporal, and perturbational context. This structure clarifies why microbiome foundation models cannot be judged only by generic prediction accuracy. Their central test is whether learned representations remain informative across cohorts, habitats, and perturbation regimes, while preserving a biologically interpretable relationship between molecular potential, ecological realization, and host or environmental context.

We therefore use the term microbiome world model as a bounded roadmap rather than as a claim that mature microbiome simulators already exist. The concept highlights a longer‐term transition from static representation learning toward models that can reason about dynamics, intervention and counterfactual ecological trajectories. At the same time, it makes explicit that such models must remain constrained by microbiome‐specific realities, including compositionality, context dependence, inter‐individual variability, incomplete observability, and the need for experimental validation.

## 3 Conceptual Foundations: What Is the “Language” of the Microbiome?

The success of transformers in biology is often framed through the “language of life” metaphor, but that metaphor must be handled with care [32, 33]. At the sequence level, it is highly productive. DNA and proteins are discrete symbol strings shaped by evolutionary and physicochemical constraints, and their local and long‐range dependencies encode motifs, domains, structural tendencies, regulatory logic, and higher‐order relationships among genes, regulatory elements, and functional modules [34, 35, 36]. In this regime, language‐modeling concepts transfer naturally: tokens are biologically meaningful, context determines interpretation, and self‐supervised objectives can uncover latent syntax and semantics without manual annotation [37, 38, 39, 40].

At the community level, however, the analogy becomes more partial [41]. Microbial communities are not intrinsically ordered symbol sequences. They are sparse ecological states, often observed as abundance profiles or presence–absence patterns, whose structure is shaped by coexistence, competition, cooperation, host environment, historical contingency, and context‐dependent interaction and regulation across taxa and functions [15, 42]. Ordering taxa by abundance or discretizing abundance states into tokens can be computationally useful, but it imposes structure that is not native to the object itself [29, 43, 44]. Sequence order is intrinsic; community order is imposed. This distinction is not philosophical decoration. It determines what can reasonably be interpreted from the learned representation [10]. A transformer trained on community tokens is not automatically learning the ecological analogue of grammar; it may instead be learning a mixture of co‐occurrence structure, habitat signatures, host‐linked gradients, regulatory coupling, interaction constraints, and technical bias.

This is why microbiome foundation models must be viewed as multiscale models from the outset [19, 45]. The field is not seeking a single universal microbiome embedding so much as a representational bridge across at least three forms of microbiome intelligence. The first is molecular intelligence, encoded in DNA and proteins and expressed as functional potential [39, 40]. The second is ecological intelligence, encoded in community composition and realized as ecological state [29, 43, 44]. The third is contextual intelligence, which emerges only when host condition, environment, time, and perturbation are incorporated [45, 46, 47]. The most interesting problems in microbiome foundation modeling arise precisely where these layers fail to align trivially.

In this Review, we define a microbiome foundation model as a model pretrained on large‐scale microbiome‐relevant data to learn reusable representations that remain informative across cohorts, technical platforms, tasks, and habitats. This definition retains the general foundation‐model criteria of large‐scale pretraining, self‐supervised or weakly supervised learning, and broad downstream adaptability, but adds a microbiome‐specific requirement: cross‐cohort and cross‐habitat transferability under ecological and technical variation. Without such transfer, a model may be useful for a specific dataset, but it should not be treated as a microbiome foundation model in the substantive sense [25, 28, 30].

## 4 Architectural and Learning Principles of Microbiome Foundation Models

Most microbiome foundation models inherit their training logic from advances in self‐supervised learning [26, 27]. Transformer encoders, autoregressive decoders, and hybrid long‐context architectures all exploit the fact that biological context can supervise representation learning without explicit labels [48, 49, 50]. Masked language modeling encourages inference of hidden sequence or community elements from surrounding context, promoting the learning of local regularities and long‐range dependencies [27, 38, 39, 51]. Autoregressive objectives support generation by training models to predict subsequent tokens or states [26, 49, 50]. Contrastive and alignment‐based objectives become important once multiple modalities, such as sequence, abundance, and host data, must be reconciled in a shared latent space [52].

Yet the decisive modeling choices often arise before architecture in the narrow sense. In microbiome research, tokenization is not just a preprocessing convenience; it is one of the central scientific decisions (Table 1). For sequence models, the choice between single nucleotides, k‐mers [38, 39], codons [53], amino acids [40], or learned sub‐sequences [51, 54] determines what biological structure the model is allowed to see. K‐mer tokenization preserves short‐range sequence syntax and remains effective for motif‐rich tasks, but it rapidly expands the vocabulary and may obscure larger units of genomic organization [38, 39]. Learned tokenization schemes compress recurrent sequence fragments and can move closer to functional units, but they may also trade away mechanistic transparency [51, 54].

**TABLE 1:** Representative microbiome and microbiome‐adjacent foundation models. The table separates mature peer‐reviewed models from emerging preprints and adjacent biological foundation models. This distinction is important because model scale alone does not establish microbiome‐level transferability, ecological validity, or robustness to cohort and habitat shift.

Model or model family	Representation and main use	Key limitations or revision note	Reference
DNABERT/DNABERT‐2; ProkBERT; Nucleotide Transformer	DNA or microbial genome sequences; transformer‐style masked or sequence modeling for taxonomic, regulatory, and functional representation.	Strong for sequence potential, but does not directly encode realized ecological activity.	[38, 39, 51, 54, 57]
Evo/Evo 2; genome‐context models	Long genomic context; genome organization, operons, gene neighborhoods and design‐oriented tasks.	Computational scale, corpus imbalance and limited host/ecological context.	[49, 50]
ESM; SaProt; protein set/genome models	Protein and protein‐set representations; structure, function, remote homology and virome‐related annotation.	Protein potential does not specify community‐level realization.	[40, 58]
FGBERT; METAGENE‐1; MetagenBERT	Emerging gene/read/metagenomic models for annotation, biosurveillance and cross‐cohort representation.	Mostly emerging or preprint evidence; needs standardized independent benchmarks.	[59, 60, 61]
MGM	Community abundance‐profile representation for ecological embedding and cross‐context prediction.	Rank‐value encoding discards absolute and relative abundance information; host‐associated pretraining imbalance may limit environmental transfer; computational biological predictions require experimental validation.	[43]
BiomeGPT	Human gut community‐profile language modeling.	Currently a bioRxiv preprint; discuss as emerging rather than peer‐reviewed benchmark evidence.	[44]
BarcodeMamba+ and pathogen‐oriented models	Fungal ITS or pathogen sequence models relevant to underrepresented microbiome components.	Adjacent to microbiome foundation modeling; community‐level ecological validation remains limited.	[62, 63]

The tokenization problem is even more revealing for community models. Microbiome communities are typically observed as continuous abundance vectors rather than discrete symbol strings [29]. Any attempt to fit them into a transformer‐style pipeline therefore imposes structure that is not biologically native [43, 44]. Rank‐value encoding privileges dominance hierarchy [43]. Abundance binning discretizes ecological continuity [44]. Taxonomy–abundance composite tokens compress several biological assumptions into a single unit. They determine whether the model is encouraged to represent relative abundance, identity, hierarchy, or interaction context, and they also determine what information is discarded.

Context length provides a second unifying principle. In genomic modeling, short‐context architectures can identify motifs but may miss long‐range regulation, operon structure, and chromosome‐scale organization [38, 39, 51]. Long‐context models attempt to extend the representational window and thereby move from local syntax toward higher‐order genomic logic [49, 50, 55]. In community modeling, an analogous issue appears not as nucleotide span but as ecological breadth: how many taxa, gradients, perturbations, and host modifiers can be represented at once [56]. In both regimes, representation design determines whether the model learns biology or memorizes dataset structure.

Pretraining offers a partial but meaningful solution. It pools information across heterogeneous corpora, reduces dependence on small labeled datasets, and often improves transfer across tasks [29, 39]. Still, pretraining should not be mistaken for biological robustness. It does not remove compositionality, eliminate batch effects, or solve annotation incompleteness. What it does provide is a stronger prior: a model begins downstream learning with an internal organization of biological regularities rather than from statistical ignorance. The key methodological question is therefore not simply whether self‐supervised learning works, but what type of biological abstraction each pretraining strategy encourages and what blind spots it leaves intact.

## 5 Sequence‐Centric Microbiome Foundation Models

**FIGURE 2:**
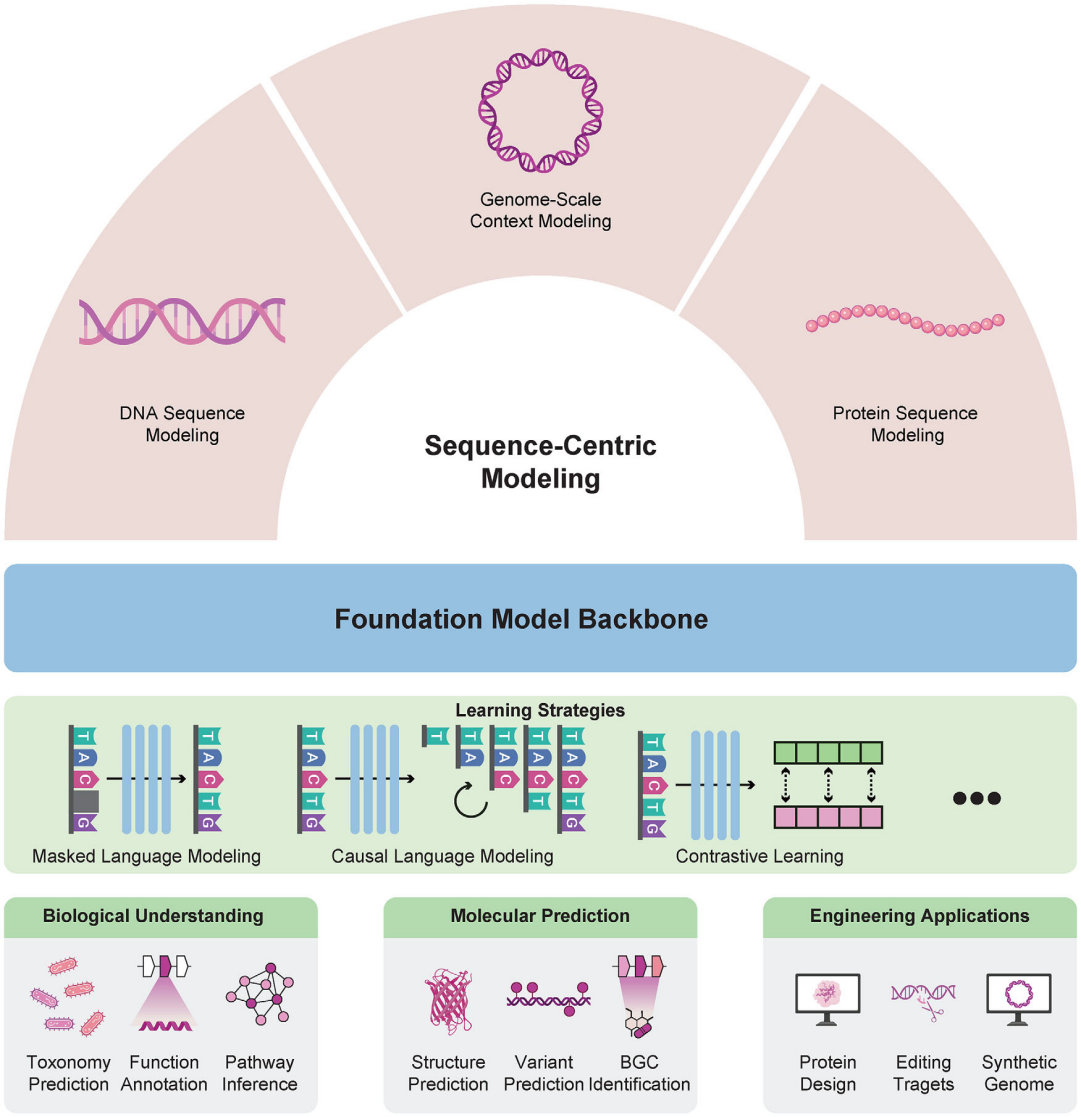
Sequence‐centric microbiome foundation models. Sequence‐centric modeling operates across multiple molecular scales, including DNA sequences, genome‐scale context, and protein sequences (top). These representations are unified through a shared foundation model backbone (middle), typically trained with self‐supervised objectives such as masked language modeling, causal language modeling, and contrastive learning. The resulting embeddings support a wide range of downstream applications (bottom), spanning biological understanding (e.g., taxonomy prediction, functional annotation, pathway inference), molecular prediction (e.g., structure prediction, variant effect prediction, biosynthetic gene cluster identification), and engineering‐oriented tasks (e.g., protein design, genome editing target discovery, and synthetic genome design).

Sequence‐centric microbiome foundation models aim to infer microbial identity and functional potential directly from molecular sequences, shifting microbiome analysis from database‐dependent annotation toward data‐driven representation learning [20]. As illustrated in Figure 2, these models operate across multiple molecular scales, including DNA sequences, genome‐scale context, and protein sequences, and are unified through a shared foundation model backbone trained with self‐supervised objectives [38, 39, 40, 49].

At the input level, sequence‐centric modeling spans three primary biological representations. DNA sequence models treat nucleotide sequences as structured token streams, enabling transformers to capture both local motifs and long‐range dependencies [38, 55]. This paradigm, exemplified by models such as DNABERT, ProkBERT, and the Nucleotide Transformer, demonstrates that genomic sequences contain reusable statistical structure that supports tasks such as regulatory element prediction, variant interpretation, and taxonomic inference [38, 39, 57]. In microbiome contexts, where sequence novelty is pervasive, this approach reframes metagenomic analysis as learning within an open sequence space rather than mapping to predefined references [3, 20].

Beyond nucleotide sequences, protein language models provide a complementary representation layer that is often more directly linked to biochemical function. Models such as ESM and SaProt encode structural and functional constraints embedded in amino acid sequences, enabling the detection of domain organization, catalytic residues, and remote homology even in the absence of close sequence matches [40, 58]. This is particularly important for microbiome datasets, where many proteins lack high‐identity homologues yet retain conserved functional roles.

A third level extends sequence modeling to genome‐scale context [64, 65, 66]. Rather than treating sequences as independent units, genome‐level foundation models incorporate gene order and genomic organization, capturing higher‐order structures such as operons, gene neighborhoods, and biosynthetic gene clusters. By integrating protein‐level embeddings with genomic context, these models shift the representational focus from molecular syntax to functional organization, better reflecting how microbial phenotypes are encoded.

These heterogeneous sequence representations are unified through a foundation model backbone (Figure 2, middle), typically implemented using transformer architectures trained with self‐supervised learning strategies. Common objectives include masked language modeling, causal language modeling, and contrastive learning, which together enable models to learn contextualized embeddings without explicit labels. This backbone serves as a general‐purpose representation engine, allowing knowledge learned at scale to be transferred across diverse downstream tasks.

The learned representations support a wide spectrum of applications (Figure 2, bottom). At the level of biological understanding, they enable taxonomy prediction, functional annotation, and pathway inference. At the molecular level, they facilitate structure prediction, variant effect prediction and biosynthetic gene cluster identification. In addition, these representations increasingly support engineering‐oriented applications, including protein design, genome editing target discovery, and synthetic genome construction. This breadth of downstream utility highlights the role of sequence‐centric models as a unifying representation layer for microbiome analysis.

Despite these advances, sequence‐centric models remain fundamentally limited to modeling functional potential rather than realized biological activity [46, 67]. While they can infer what a genome or protein is capable of, they provide limited insight into when and under which ecological conditions these functions are expressed. Microbial behavior is shaped by community interactions, host environment, and external perturbations, factors that are not explicitly encoded in sequence data alone [68]. Furthermore, sequence models inherit biases from pretraining corpora, including phylogenetic imbalance and uneven annotation density.

Taken together, sequence‐centric foundation models constitute a foundational layer of microbiome representation learning (Figure 2), providing a scalable and generalizable backbone for molecular understanding. However, capturing microbiome dynamics and ecological function will require extending beyond sequence representations toward community‐level and context‐aware modeling.

Phage, virome, fungal, and pathogen‐focused modeling remain underrepresented in current microbiome foundation‐model discussions. Protein‐set and viromics‐oriented models can help annotate high‐diversity viral genomes, while fungal ITS barcode models and pathogen‐oriented DNA foundation‐model adaptations illustrate how non‐bacterial microbiome components may require distinct tokenization and evaluation schemes. These directions should be described as emerging or adjacent unless they demonstrate transfer across microbiome cohorts and habitats [62, 63, 65].

## 6 Community‐Centric Foundation Models

**FIGURE 3:**
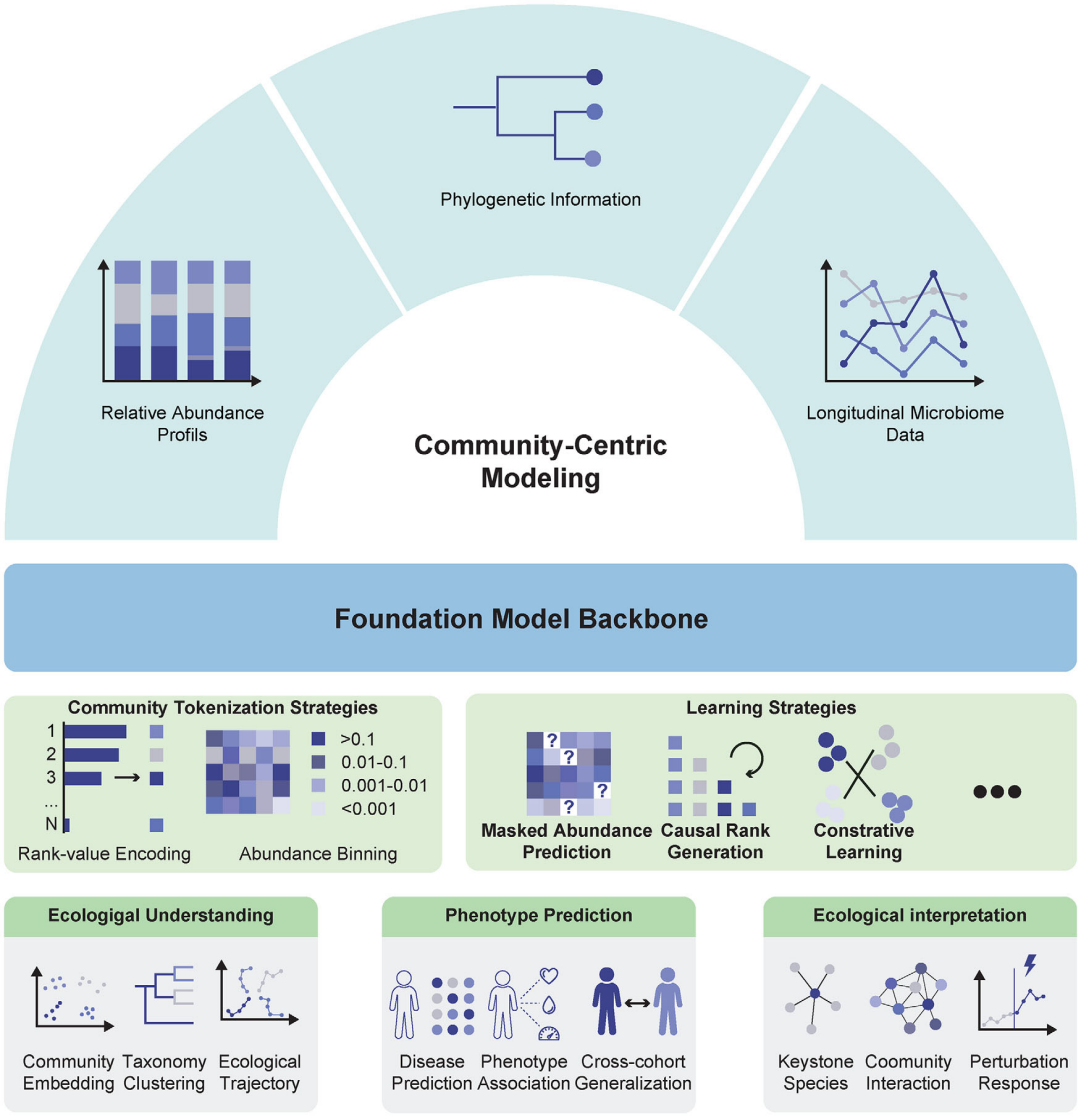
Community‐centric microbiome foundation models. Community‐centric modeling operates on ecological‐level representations of microbial communities, including relative abundance profiles, phylogenetic information, and longitudinal microbiome data (top). These heterogeneous inputs are unified through a shared foundation model backbone (middle), which relies on explicit community tokenization strategies to convert non‐sequential ecological data into model‐compatible representations. Such strategies include rank‐value encoding and abundance binning, enabling the transformation of compositional profiles into structured inputs. Model training is typically driven by self‐supervised learning objectives (middle), including masked abundance prediction, causal rank generation, and contrastive learning, allowing the model to learn latent ecological state embeddings without labeled data. The learned representations support diverse downstream applications (bottom). At the ecological level, they enable community embedding, taxonomy‐aware clustering, and trajectory analysis. For predictive tasks, they facilitate disease prediction, phenotype association, and cross‐cohort generalization. In addition, these models support ecological interpretation, including keystone species identification, community interaction inference, and perturbation response modeling. Despite their effectiveness, community‐centric models depend on manually designed tokenization schemes, reflecting the lack of intrinsic sequential structure in microbial communities and highlighting a key limitation of current ecological representation learning approaches.

Community‐centric foundation models operate at the ecological level, shifting the modeling focus from molecular encoding to community organization across samples, hosts, and environments [29, 43, 44]. As illustrated in Figure 3, these models take heterogeneous ecological inputs, including relative abundance profiles, phylogenetic information, and longitudinal microbiome data, and aim to learn transferable representations of community state.

Unlike sequence‐centric approaches, these inputs lack an intrinsic sequential structure [41]. As a result, a central design component of community‐centric modeling lies in transforming compositional data into model‐compatible representations [29]. This is typically achieved through explicit tokenization strategies (Figure 3, middle; Table 2), such as rank‐value encoding and abundance binning, which impose an artificial ordering or discretization over taxa abundance [43, 44]. These transformations enable the use of foundation model backbones but also introduce an additional layer of inductive bias that shapes what the model can learn.

**TABLE 2:** Comparison table of different community tokenization methods.

Strategy	Inductive bias	Strength	Main limitation	Best‐suited evaluation
Rank‐value encoding	Dominance hierarchy is treated as informative order	Captures relative dominance and common ecological gradients	Sensitive to ties, rare taxa, and compositional perturbations	Permutation sensitivity; cross‐cohort transfer
Abundance binning	Continuous abundance is discretized into ecological states	Reduces noise and enables masked‐token objectives	May discard fine abundance gradients and threshold‐sensitive signals	Calibration and perturbation robustness
Taxonomy‐aware composite tokens	Identity, hierarchy, and abundance are fused	Retains phylogenetic/taxonomic context	Can hard‐code taxonomy bias and obscure functionally convergent taxa	Cross‐habitat transfer and ecological interpretation consistency

Community tokenization is not a neutral preprocessing step. Because microbial communities do not have an intrinsic sequence order, every transformer‐compatible representation imposes a modeling hypothesis about what should count as context. The key question is therefore not only whether a tokenization improves downstream accuracy, but what ecological assumption it builds into the representation and whether that assumption remains valid across cohorts and habitats.

Operationally, community tokenization should be evaluated by permutation sensitivity, robustness to compositional and zero‐inflation perturbations, cross‐cohort and cross‐habitat transfer, calibration under perturbation prediction, and consistency of ecological interpretation. A model that performs well only under one imposed ordering may be learning dataset structure rather than transferable ecological organization.

Once tokenized, community profiles are processed through a shared foundation model backbone (Figure 3), typically based on transformer architectures trained with self‐supervised learning objectives. Common learning strategies include masked abundance prediction, which captures co‐occurrence structure; causal rank generation, which models compositional ordering; and contrastive learning, which aligns samples across cohorts or conditions. Together, these objectives enable models to learn latent ecological embeddings without requiring explicit labels.

The resulting representations support a broad range of downstream applications (Figure 3, bottom). At the ecological level, they enable community embedding, taxonomy‐aware clustering, trajectory analysis, and context‐aware ecological comparison across longitudinal samples. In predictive settings, they facilitate disease prediction, phenotype association and cross‐cohort generalization. In addition, they provide a basis for ecological interpretation, including the identification of keystone species, inference of community interactions, characterization of context‐dependent ecological structure, and modeling of perturbation responses. Models such as MGM and BiomeGPT exemplify this paradigm by leveraging large‐scale microbiome datasets to learn reusable ecological representations across diverse environments.

Recent community‐centric models should therefore be interpreted in light of model‐specific limitations. MGM [43] converts^1^ abundance tables into sequential representations using rank‐value encoding, which mitigates the influence of extreme values but sacrifices absolute and relative abundance information. Hybrid encoding could combine rank‐based robustness with abundance‐preserving features. Its pretraining corpus is also ecologically imbalanced, with more than 60% of MGnify entries derived from host‐associated microbiomes [43]. This imbalance may bias learned representations toward host‐associated patterns and weaken transfer to soil, marine, and engineered ecosystems. Finally, its proposed treatment targets and keystone genera are supported primarily by computational analyses and require perturbation‐based experimental validation.

BiomeGPT [44], by contrast, is currently available as a bioRxiv preprint and should therefore be treated as an emerging example rather than a peer‐reviewed benchmark model. Its evaluation should prioritize independent cross‐cohort and cross‐habitat tests, comparisons with conventional microbiome machine‐learning baselines, and explicit reporting of failure cases.

However, these advances come with a fundamental conceptual limitation [15]. Community‐centric foundation models are, in essence, sequence‐shaped architectures applied to ecological objects that are not inherently sequential. While tokenization enables the application of powerful representation learning methods, it does not guarantee that the learned structure corresponds to true ecological mechanisms. Dependencies inferred between taxa may arise from direct interactions, shared environmental constraints, host‐associated covariation, or technical artifacts. Consequently, representational structure should not be directly interpreted as causal ecological relationships.

This limitation is further reflected in the challenge of capturing ecological conditionality. Microbial roles are highly context‐dependent, varying across environmental conditions, host states, and community compositions. Static or weakly contextualized embeddings may therefore underrepresent the dynamic nature of microbial function. Although recent models partially address this by conditioning on sample context or incorporating longitudinal data, they do not fully resolve the mismatch between imposed sequential representations and inherently compositional, context‐dependent ecological systems.

Taken together, community‐centric foundation models represent both a significant methodological advance and a clarification of the field's current limitations (Figure 3). They provide a scalable framework for encoding community‐level variation and enable strong performance across a range of predictive and interpretive tasks. At the same time, they highlight that abundance‐based representations alone are insufficient for mechanistic understanding, motivating the integration of additional modalities and the development of models that more faithfully capture ecological dynamics and causality.

## 7 Toward Integrated and Multimodal Microbiome Foundation Models

**FIGURE 4:**
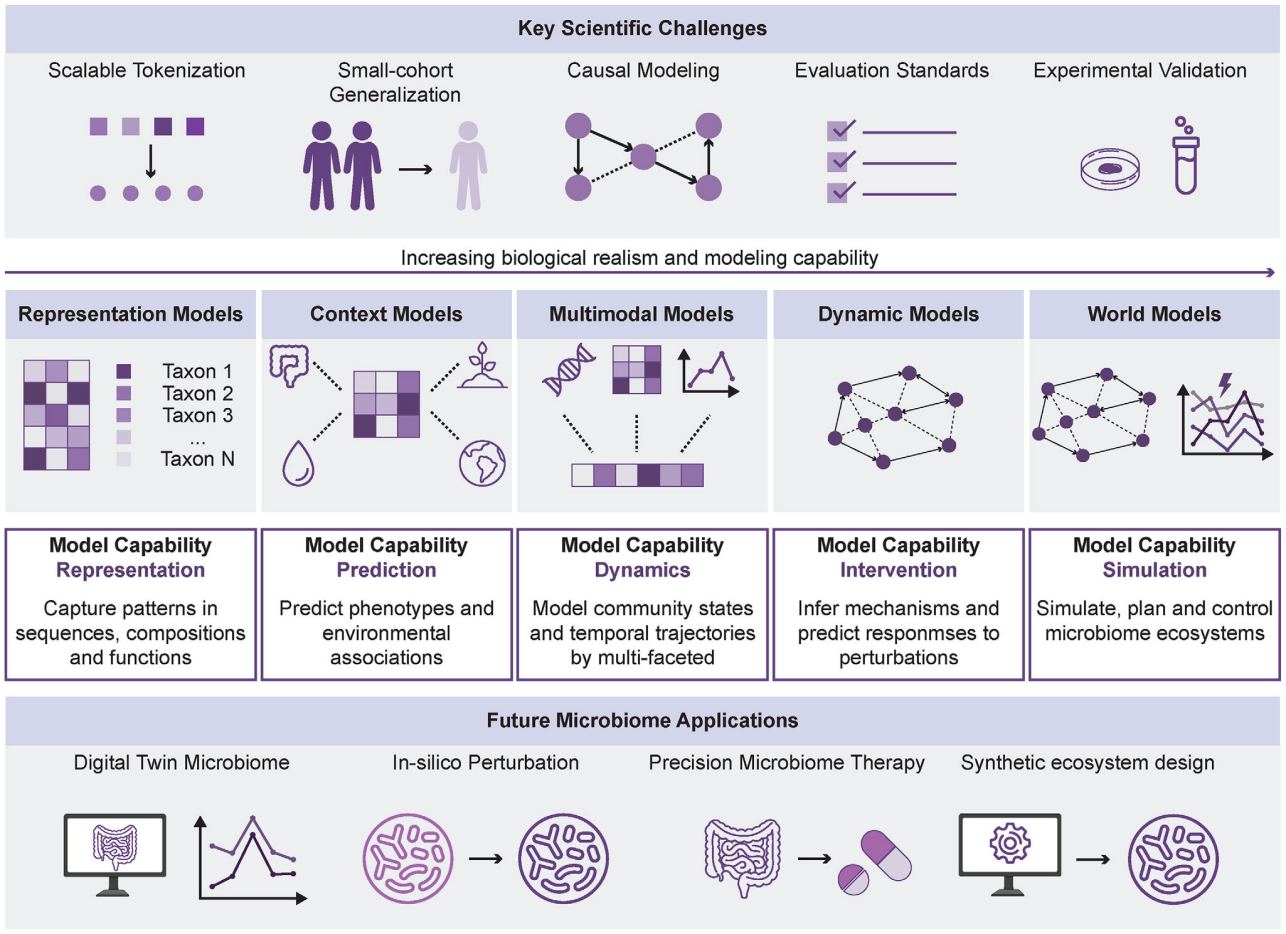
From representation to world models: a roadmap for microbiome foundation modeling. Microbiome foundation models are evolving along a conceptual trajectory from representation models to world models (middle), with progressively increasing modeling capability from representation and prediction to dynamics, intervention, and simulation. Early‐stage models focus on encoding community structure, followed by context‐aware models that incorporate host and environmental factors, and multimodal models that integrate heterogeneous biological signals, including transcriptomic, proteomic, metabolomic, spatial, and phenotype‐level information. More advanced dynamic models explicitly incorporate time, temporal dependence, and perturbation history, enabling the modeling of trajectories, resilience, and responses to interventions such as antibiotics, diet, and microbial therapeutics, and ultimately leading toward world models capable of simulating ecosystem behavior under alternative futures. This progression is accompanied by key scientific challenges (top), including scalable tokenization of microbiome data, generalization across small and heterogeneous cohorts, causal modeling of microbial interactions, the establishment of standardized evaluation frameworks, and the need for experimental validation. At the application level (bottom), these advances enable emerging capabilities such as digital twin microbiomes, in silico perturbation testing, precision microbiome therapy and synthetic ecosystem design. Together, this framework outlines a transition from association‐based modeling toward predictive, controllable and mechanistic microbiome intelligence.

The distinction between sequence‐centric and community‐centric microbiome modeling is analytically useful but biologically incomplete [45]. Microbial systems are inherently multi‐layered, spanning genomic potential, community organization, host interaction, and environmental context [19, 21, 46]. As illustrated in Figure 4, microbiome foundation modeling is undergoing a conceptual transition from isolated representation learning toward integrated, multimodal frameworks that align these complementary perspectives [52].

The transition to multimodal microbiome foundation models is not a simple matter of concatenating embeddings. Sequence‐centric models primarily represent functional potential, whereas community‐centric models represent ecological state under a particular host, habitat, and technical context. These feature spaces may be statistically correlated without being biologically aligned. In practice, alignment must be tested by matched multi‐omics data, perturbational trajectories, and biological‐consistency checks rather than by latent‐space proximity alone.

Current alignment strategies, including contrastive learning and cross‐modal attention, are limited by sparse paired measurements, batch effects, host and environmental confounding, missing temporal information, and incomplete functional annotation. A shared embedding that improves prediction can still fail to preserve causal or ecological meaning if the paired modalities encode different sampling biases.

A central organizing principle in this transition is the distinction between functional potential and ecological realization. Sequence‐centric models estimate what microbial genomes and proteins could encode, whereas community‐centric models describe how microbial assemblages are structured under specific conditions. However, neither layer alone explains how microbial functions are realized in situ. This depends on additional contextual factors, including host physiology, environmental conditions, and temporal dynamics.

Multimodal microbiome foundation models emerge as a natural solution to this gap. By integrating sequence‐derived features, community composition, host‐associated signals and, increasingly, additional molecular and phenotype‐level modalities, these models begin to constrain biological interpretation across scales. In this framework, molecular representations inform ecological structure, while ecological context provides constraints on functional inference. Such cross‐scale alignment enables more robust generalization, particularly in settings involving novel taxa or under‐sampled environments.

This integration also extends to host–microbiome coupling [46, 69]. Clinical metadata, immune state, metabolite profiles, proteomic state, spatial context, and treatment history all influence whether microbial capacity becomes realized phenotype [47, 68]. Modeling the microbiome as part of a coupled host–microbe system therefore represents a necessary step toward biologically meaningful prediction, and ultimately points toward microbiome foundation models that unify sequencing, multi‐omics and phenomics within a shared representational framework.

Spatial metatranscriptomics is not simply an additional omics channel: spatial coordinates define neighborhood structure and microhabitat‐specific context that foundation models must represent explicitly [69]. Useful representations may therefore need to encode multiscale host–microbe neighborhoods rather than treating spots or cells as exchangeable tokens. Key challenges include sparse and uneven coverage, platform‐dependent resolution, mixed host–bacterial–fungal signals, and the need to distinguish physical proximity from functional interaction. Pretraining and evaluation should consequently test neighborhood reconstruction, transfer across tissue regions, and preservation of biologically meaningful microhabitat embeddings.

## 8 From Representation to Dynamics: Expanding Modeling Capabilities

The evolution of microbiome foundation models can be understood as a progression of modeling paradigms with increasing biological realism (Figure 4). Early representation models focus on encoding microbial composition or sequence structure into latent spaces. Context models extend this by incorporating environmental and host‐associated variables, enabling more robust predictive performance. Multimodal models further integrate heterogeneous biological signals into unified representations (Table 3).

**TABLE 3:** Worked examples of microbiome foundation‐model applications.

Application	Input evidence	Potential contribution	Current caveat
Cross‐cohort disease prediction	Multi‐study abundance or metagenomic profiles	Tests whether representations transfer beyond a single cohort	Must compare against classical baselines and external cohorts.
Metagenomic/pathogen monitoring	Wastewater, environmental or clinical metagenomic reads	Supports broad sequence representation for surveillance	Mostly sequence‐level signal; ecological interpretation remains limited.
Functional annotation and novel gene discovery	Genes, proteins, contigs or genome neighborhoods	Improves annotation in reference‐poor microbiome sequence space	Functional potential does not guarantee in situ activity.
Perturbation and digital‐twin prediction	Longitudinal samples with intervention records	Supports in silico comparison of alternative futures	Requires dense initialization, calibration and experimental validation.

Beyond static modeling, dynamic models introduce temporal structure and begin to capture ecological interactions and trajectories [42, 56, 70, 71]. These models move beyond cross‐sectional prediction to represent how microbial communities evolve over time. This shift is essential for distinguishing transient perturbations from stable ecological states and for modeling resilience, recovery, and instability.

Correspondingly, model capabilities expand from representation and prediction toward dynamics, intervention and simulation (Figure 4) [72]. While current models are primarily optimized for phenotype prediction, future systems should explicitly model time, conditional trajectories and responses to perturbations [67]. This includes predicting the effects of antibiotics, dietary shifts, or therapeutic interventions, and comparing alternative strategies in silico.

Temporal and perturbational data play a critical role in enabling this transition [56, 72]. Static snapshots are insufficient to capture the dynamical nature of microbial ecosystems. Longitudinal measurements, explicit perturbation records, and intervention‐aware modeling provide the basis for learning causal and time‐dependent relationships. This marks a fundamental shift from association‐based modeling toward systems‐level understanding.

## 9 Key Scientific Challenges in Microbiome Foundation Modeling

Despite rapid progress, several fundamental challenges continue to constrain the field (Figure 4; Table 4). First, scalable and biologically meaningful tokenization remains unresolved. Sequence tokenization, abundance discretization, and multimodal representation schemes define what the model can learn, yet many current approaches are driven more by architectural convenience than biological principle.

**TABLE 4:** Data resources and infrastructure needs for microbiome foundation models.

Resource type	Examples to verify	Use in foundation modeling	Key infrastructure need
Sequence and metagenomic archives	MGnify, SRA/ENA‐derived corpora, UHGG	Pretraining sequence, gene, and genome‐context models	Clear licenses, deduplication, habitat metadata, and contamination control.
Human microbiome cohorts	HMP/iHMP, MetaCardis, curatedMetagenomicData‐like resources	Community representation and cross‐cohort evaluation	Standardized phenotype, protocol, and batch metadata.
Virome and fungal resources	Virome catalogs, fungal ITS/barcode datasets	Coverage of underrepresented microbiome kingdoms and viruses	Better taxonomic curation and ecological context.
Longitudinal and perturbational cohorts	Diet, antibiotic, probiotic, disease‐flare and intervention studies	Dynamics, resilience and digital‐twin evaluation	Dense time series, intervention timing and host/environmental covariates.
Paired multi‐omics cohorts	Metagenome, metatranscriptome, metabolome, proteome and spatial datasets	Multimodal alignment and biological‐consistency testing	Matched sampling, shared identifiers, and open benchmark splits.

Second, generalization across small and heterogeneous cohorts remains limited. Microbiome data are highly variable across studies due to differences in sampling, sequencing, host populations, and environments. Large‐scale pretraining can mitigate but not eliminate domain shift. The field's progress will depend not only on model scale, but also on open and auditable data infrastructure. Pretraining corpora, benchmark splits, metadata standards, model weights, and evaluation code should be made as accessible as ethical, legal, and consent constraints allow [3, 4, 73].

Third, causal modeling remains a central challenge. Observed associations between taxa may reflect direct interactions, shared environmental drivers, or host‐mediated effects. Without controlled perturbation data, distinguishing these mechanisms is inherently difficult.

Fourth, evaluation standards are underdeveloped. Many benchmarks emphasize cross‐sectional predictive accuracy, which does not necessarily reflect biological validity or robustness. More informative evaluation frameworks should assess transferability, calibration, stability, and the ability to predict responses to perturbation.

Finally, experimental validation represents a major bottleneck. Unlike text or image generation, microbiome predictions often require labor‐intensive validation through culture, perturbation experiments, or in vivo studies. This limits the speed at which model‐driven hypotheses can be confirmed.

A hierarchical view of these challenges is useful. Conceptual barriers include compositionality, imposed community order, and causal identification. Methodological barriers include tokenization, domain shift, multimodal alignment, and benchmark design. Practical barriers include cohort size, metadata heterogeneity, restricted data access, limited model openness, and slow experimental validation. These levels are interdependent: causal modeling depends on perturbation data, benchmark reliability depends on standardized metadata, and generalization claims depend on cross‐cohort and cross‐habitat tests.

Benchmarking should move beyond cross‐sectional predictive accuracy. We recommend standardized evaluation along at least six dimensions: cross‐cohort transferability, cross‐habitat generalization, robustness to batch effects and zero‐inflation perturbations, calibration of perturbation predictions, stability of ecological interpretation, and comparison with conventional microbiome statistical or machine‐learning baselines. Recent robustness studies of tabular foundation models on microbiome‐like data reinforce the need to evaluate distribution shift explicitly rather than assuming that foundation‐model scale guarantees robustness [74].

## 10 Toward Microbiome World Models: Intervention and Simulation

The long‐term trajectory of microbiome foundation modeling points toward the development of microbiome world models (Figure 4). In the broader artificial intelligence literature, world models refer to learned internal models of environment dynamics that support prediction, counterfactual rollout, and action selection in latent space [75, 76, 77]. Unlike current models that primarily describe capacity or state, world models aim to represent how microbial systems evolve under intervention and to support counterfactual reasoning.

This distinction is particularly important for microbiome research. Microbial communities are intrinsically ecological systems that are only partially observable, highly context dependent, and continuously reshaped by interactions among microbes, the host, and the environment. Community composition therefore provides only a static description of system state, whereas predicting the consequences of perturbations requires modeling latent ecological dynamics, feedback loops, and temporal adaptation. These characteristics motivate a microbiome‐specific interpretation of world models as internal representations of microbial ecosystem dynamics rather than direct adaptations of existing AI world‐model frameworks.

Single‐cell transcriptomic atlases and protein‐interaction networks are often analyzed as molecular‐state snapshots or relatively static relational maps. By contrast, longitudinal microbiome studies can capture reciprocal ecological feedback, rapid community transitions, and responses to tractable system‐level perturbations such as antibiotics and diet. These features create both a stronger need and, in well‐controlled longitudinal settings, a practical route to the intervention‐response data required by world models.

Such models are inherently dynamical. They must operate over trajectories rather than snapshots and reason about conditional futures rather than static embeddings. Perturbations such as antibiotics, dietary changes, immune modulation, or microbial therapies reshape the microbiome through coupled host–microbe and microbe–microbe interactions. Although the term microbiome world model is still emerging, several adjacent lines of research already instantiate key components of this paradigm, including digital twin modeling, ecosystem‐scale dynamical inference, perturbation‐aware prediction and temporal graph‐based forecasting [71, 72, 78, 79]. Capturing these processes requires integrating sequence‐level capability, community structure, host context, and temporal memory within a unified framework.

One emerging realization of this concept is the microbiome digital twin. A digital twin seeks to approximate an individual microbial ecosystem sufficiently well to support in silico experimentation, hypothesis testing, and intervention planning. Early studies suggest that such systems may enable the screening of therapeutic strategies, optimization of probiotic design, and prediction of ecological responses prior to empirical validation [72]. Current examples remain narrow and application‐specific, but they demonstrate that individual‐level microbiome simulation is becoming technically plausible rather than purely aspirational [80].

The feasibility boundary of microbiome digital twins should therefore be stated explicitly. Current systems are most plausible for constrained settings with dense longitudinal sampling, limited ecological complexity, and well‐characterized perturbations. Scaling from infant or narrowly defined microbiomes to complex adult ecosystems will require extensive initialization data, repeated host and environmental measurements, matched multi‐omics, and model architectures that can control error accumulation during long counterfactual rollouts. Without these constraints, digital twins risk becoming overconfident simulators of unobserved ecological futures rather than experimentally accountable models.

In parallel, generative microbiome engineering represents another frontier. Sequence‐level generators may design proteins, pathways, or phages, whereas community‐level models may guide the construction of synthetic consortia with desired functional properties. However, the central challenge is not generation alone, but generation under ecological and physiological constraints.

Taken together, microbiome world models should be viewed as a long‐term research roadmap rather than a mature modeling paradigm. Their central objective is to move beyond association‐based prediction toward predictive, controllable, and mechanistic reasoning about microbial ecosystems. Realizing this vision will require advances not only in model architecture, but also in experimental integration, causal inference, and evaluation frameworks. Rather than implying that general‐purpose microbiome simulators already exist, we argue that current foundation models constitute important building blocks toward future systems capable of intervention reasoning, simulation, and microbiome design.

## 11 Conclusions

Foundation models are changing microbiome research by changing its unit of intelligence. Instead of treating microbiome data primarily as cohort‐specific feature tables, they make it possible to learn representations that span genes, proteins, genomes, taxa, communities and host‐linked states. So far, however, most microbiome foundation models remain centered on sequencing‐derived inputs, especially DNA sequences, abundance profiles and related molecular readouts. The next stage will require broader multimodal integration across transcriptomics, proteomics, metabolomics, spatial measurements and phenotype‐level data, so that models can represent not only microbial potential and composition, but also realized host‐associated function. This shift has already advanced sequence‐based function inference, ecological state representation and multimodal integration. More importantly, it has made visible a deeper scientific ambition: to represent microbial systems across scales rather than only to associate them with labels.

The field now stands at a critical transition. Sequence‐centric and community‐centric models have each demonstrated both power and insufficiency. One captures functional potential without ecological realization; the other captures ecological state without molecular mechanism. The most important advances will therefore come from models that align these layers while explicitly confronting compositionality, domain shift, evaluation, interpretability, and validation. More comprehensive microbiome foundation models will also need to integrate multiple omics together with host physiology, clinical metadata and phenomics, moving beyond sequencing‐only abstraction toward a fuller representation of microbial system state.

The endpoint of microbiome foundation modeling is not simply a larger model or a more accurate classifier. It is a biologically grounded, intervention‐aware framework for reasoning about microbial systems as dynamical entities. In this sense, microbiome world models should be understood not as an isolated microbiome‐specific idea, but as part of a broader shift in artificial intelligence from static pattern recognition toward learned dynamical models that support prediction, simulation, and decision‐making. Whether the field reaches that goal will depend less on raw scale than on its ability to turn representation learning into mechanistic and experimentally accountable microbiome science.

A practical research roadmap should be time‐stratified. In the near term (1–3 years), the highest priorities are transparent model comparison, community‐tokenization evaluation, cross‐cohort and cross‐habitat benchmarks, and better reporting of pretraining data and model openness. In the mid term (3–5 years), progress should focus on multimodal alignment, longitudinal perturbation modeling, calibrated ecological interpretation, and experimentally grounded validation. In the long term (>5 years), the field may move toward intervention‐aware microbiome world models, digital twins and closed‐loop microbiome engineering, but only if simulation claims remain constrained by measured dynamics, uncertainty estimation and experimental feedback.

## Author Contributions

H.Z. and Z.K. drafted the manuscript and prepared the figures. K.N. conceived and supervised the study, and critically revised and proofread the manuscript.

## Conflicts of Interest

Haohong Zhang and Kang Ning are authors of the MGM study cited in this Review. This relationship is disclosed in a footnote at the first substantive discussion of MGM. The authors declare no other competing interests.

## Data Availability

Data sharing not applicable to this article as no datasets were generated or analysed during the current study.

## Footnotes

1 MGM was developed by Haohong Zhang and Kang Ning, authors of this Review. The model is described from the published report and assessed using the same critical criteria applied to other models.
